# Seasonal Phenology and Developmental Potential of *Halyomorpha halys* Under Temperate Continental Conditions

**DOI:** 10.3390/biology15161397

**Published:** 2026-08-14

**Authors:** Martina Pajač Beus, Darija Lemić, Aleksandar Mešić, Ivana Pajač Živković

**Affiliations:** Department of Agricultural Zoology, Faculty of Agriculture, University of Zagreb, Svetošimunska 25, 10000 Zagreb, Croatia; mpajacbeu23@student.agr.hr (M.P.B.); amesic@agr.hr (A.M.); ipajac@agr.hr (I.P.Ž.)

**Keywords:** brown marmorated stink bug, seasonal development, degree-days, phenology, overwintering survival, pheromone trapping, integrated pest management

## Abstract

The seasonal development and overwintering success of *Halyomorpha halys* remain insufficiently resolved in temperate continental regions of southeastern Europe, where climatic conditions may permit more than one generation per year. We monitored seasonal development, thermal accumulation, overwintering survival, and pheromone-trap captures of *H. halys* during 2024 and 2025 in continental Croatia. Experimental populations were maintained under unheated semi-outdoor conditions and followed from overwintered adults through oviposition, all five nymphal instars, and adult emergence. Degree-days were calculated above a lower developmental threshold of 12.2 °C. Two complete egg-to-adult generations developed in both years. The observed phenological interval from the first egg masses to first adult emergence was 56 days in 2024 and 42 days in 2025 for G1, and 49 and 53 days, respectively, for G2. Thermal accumulation during immature development ranged from 468.5 to 562.7 DD_12.2_. First-generation adults emerged in early July in both years, whereas second-generation adults appeared on 12 September 2024 and 23 September 2025. Developmental stages overlapped during summer and early autumn. Overwintering survival under the two anthropogenic refuge conditions was low, reaching 9.4% under heated and 7.8% under unheated indoor conditions. Pheromone-trap catches showed marked seasonal variation but were not correlated with temperature, relative humidity, or precipitation during corresponding trapping intervals. These results demonstrate the developmental potential for two generations under temperate continental conditions in Croatia and provide phenological benchmarks for stage-specific monitoring. Monitoring should begin with spring adult activity and be intensified during May–June and again from late July onwards as successive generations develop.

## Simple Summary

The seasonal biology of the invasive brown marmorated stink bug, *Halyomorpha halys*, is strongly shaped by local climatic conditions and determines the periods when crops are exposed to different developmental stages. This study investigated seasonal development, thermal accumulation, overwintering survival, and adult activity of *H. halys* under temperate continental conditions in Croatia. By following successive generations separately, we confirmed that two complete developmental cycles could be achieved within a single growing season under the conditions recorded during both study years. First-generation adults appeared in early July, while the second generation developed during late summer and produced adults in September. Considerable temporal overlap among eggs, nymphal instars, and adults demonstrated that several developmental stages may coexist during important periods of crop exposure. Overwintering survival was low under the two anthropogenic refuge conditions examined, indicating that winter survival may represent an important seasonal demographic constraint. Pheromone-trap captures complemented the cohort observations by describing seasonal adult activity. Together, these findings provide a locally based phenological framework for interpreting *H. halys* seasonal dynamics and identify periods when stage-specific monitoring should be intensified to support more precisely timed integrated pest management.

## 1. Introduction

Native to East Asia, the brown marmorated stink bug, *Halyomorpha halys* (Stål, 1855), has become established across North America and Europe [1,2,3,4]. The species is now an important agricultural pest and a nuisance in urban and residential environments, with considerable consequences for crop production [2,5]. Its establishment across a wide range of environments is facilitated by its broad host range, high dispersal capacity, and ability to adjust life-history traits to local environmental conditions [6,7,8,9,10].

The seasonal biology of *H*. *halys* is strongly influenced by temperature and photoperiod, which regulate development, reproduction, and diapause induction [11,12,13,14]. Temperature-dependent development affects survival, fecundity, and population growth [15,16,17], while invasive populations can adjust their seasonal development to local environmental conditions [18,19]. Thermal accumulation, expressed as degree-days, determines the potential number of generations, ranging from univoltinism in cooler climates to bivoltinism or polyvoltinism in warmer regions [3,20,21]. Host plant availability and landscape structure also affect seasonal population dynamics [22,23,24,25,26].

Overwintering is an important demographic bottleneck because the proportion of adults surviving winter determines the reproductive population in the following season [27,28,29]. Adults enter facultative diapause in response to environmental cues [13,30] and seek sheltered microhabitats, where survival depends on microclimatic conditions and physiological reserves [31,32,33,34]. The timing of post-diapause emergence and subsequent movement to host plants also affects seasonal population development [35,36]. These seasonal shifts in behaviour and habitat use must also be considered when interpreting monitoring data.

Interpretation of pheromone-trap captures requires caution because catches do not necessarily provide a direct measure of absolute population abundance. Trap captures result from the interaction between insect movement, detection of the attractant, and physiological motivation [37,38]. During movement towards overwintering sites, increased shelter-seeking behaviour may produce late-season peaks in trap catches that are not proportional to changes in population size [38]. Regional trap averages may also obscure local differences in developmental timing and population intensity. In Emilia-Romagna, Italy, stage-specific peaks followed a consistent sequence, but correlations between consecutive developmental stages were only moderate, indicating spatial variation among monitoring sites [39].

This limitation also affects the use of trap captures for treatment decisions. Short et al. [40] proposed a provisional threshold of 10 cumulative adults per pheromone-baited trap in apple orchards, which provided fruit protection while reducing insecticide applications by approximately 40%. However, this threshold was developed for a specific crop and production system, and the relationship between trap captures and crop injury may vary with trap placement, adjacent habitats, and crop type [41,42]. A broadly applicable treatment threshold for *H*. *halys* has therefore not yet been established.

In Croatia, *H*. *halys* was first recorded in 2017 [43]. Following its initial detection in Rijeka, the species spread into continental Croatia. Its first mass occurrence in agricultural production was recorded in 2019 in a soybean field at Drenčec, near Zagreb, where 723 nymphs and adults were collected, corresponding to an average density of 14 individuals per 10 plants [44]. This finding indicated the establishment of a locally abundant population in central Croatia and a potential risk to soybean and other crops. Subsequent pheromone-based monitoring in perennial crops detected *H*. *halys* in all surveyed Croatian regions and host crops. In an apple orchard in Zagreb County, weekly captures reached 11 adults and exceeded the provisional threshold proposed by Short et al. [40] and Pajač Živković et al. [45].

More recent research provided the first quantitative evidence of economically relevant fruit damage caused by *H*. *halys* in a commercial apple orchard in northwestern Croatia. During two consecutive seasons, damage incidence and estimated potential yield losses differed between cultivars. In ‘Cripps Pink’ apples, estimated potential yield losses reached 29% in 2024 and 69% in 2025, whereas the corresponding values for ‘Fuji’ apples were 13% and 5%, respectively [46]. Damage in ‘Cripps Pink’ occurred mainly in the middle and upper canopy layers and was higher in parts of the orchard adjacent to the forest edge, whereas damage in ‘Fuji’ remained lower and more evenly distributed. Affected fruits also showed cultivar-dependent changes in size, skin colour, and physicochemical properties. These results indicate that the impact of *H*. *halys* depends not only on population abundance but also on cultivar susceptibility and the spatial distribution of injury within the orchard [46].

The first mass occurrence near Zagreb, subsequent captures above the provisional treatment threshold, and the documentation of potential yield losses in a commercial apple orchard show the increasing agricultural importance of *H*. *halys* in continental Croatia. However, information on its seasonal development and overwintering capacity under temperate continental conditions remains limited.

In northern Italy, an outdoor life-table study under temperate–Mediterranean climatic conditions showed that *H*. *halys* can complete two generations per year [20]. The study also showed that mortality during overwintering and the post-diapause period may reduce the number of individuals contributing to reproduction in the following season. In western Slovenia, a three-year outdoor study under sub-Mediterranean conditions also recorded two overlapping generations per year [47]. Adults of the first new generation began to emerge in mid-summer, whereas adults of the second generation appeared from mid-September onwards and entered reproductive diapause without producing an additional generation. Development from egg to adult required approximately 531 degree-days for the first generation and 545 degree-days for the second generation above a lower developmental threshold of 12.2 °C. Mortality from the beginning of overwintering to the subsequent reproductive period ranged from 71.2% to 80.8% among years [47]. A different pattern was reported in the urban landscape of central Slovenia. Based on three years of pheromone-trap monitoring at two locations in Ljubljana, Bohinc et al. [48] concluded that *H*. *halys* completed one generation per year under continental conditions. Overwintered adults were generally detected during the first half of April, nymphs appeared from late June or early July onwards, and adults of the new generation were recorded mainly from late August to late September. Using a lower developmental threshold of 12.2 °C, overwintered adults were first recorded at approximately 1–11 accumulated degree-days, young nymphs at 290–516 degree-days, older nymphs at 290–812 degree-days, and new-generation adults at approximately 809–1204 degree-days, depending on year and location. Average daily temperature and precipitation were used together with degree-day accumulation to interpret the seasonal occurrence of developmental stages [48]. Three years of pheromone-based monitoring in kiwifruit orchards in northern Greece indicated seasonal patterns consistent with two overlapping generations per year [49]. Overwintered adults became active in spring, and the seasonal sequence of adult and second-instar nymph captures was consistent with the development of two generations. The timing and magnitude of population peaks varied among regions and years, indicating that local environmental conditions affected the observed phenological pattern [49].

These studies show regional differences in the seasonal biology of *H*. *halys* in southern and central Europe, ranging from univoltinism under cooler continental conditions to bivoltinism in warmer sub-Mediterranean and Mediterranean environments. Comparisons among studies should also consider differences in methodology, because some studies followed defined cohorts under outdoor conditions, whereas others inferred generational patterns from pheromone-trap captures and the seasonal occurrence of developmental stages. No previous study has documented the full seasonal development of *H*. *halys* over successive years under the temperate continental conditions of Croatia.

The aim of this study was to document the seasonal development of *H*. *halys* over two consecutive years in continental Croatia, and to assess whether the species completes more than one generation per year under temperate continental conditions. Adult overwintering survival was also evaluated under semi-natural outdoor and heated indoor conditions. Given the recently documented cultivar-dependent damage and potential yield losses in Croatian apple production, improved knowledge of seasonal development is needed to identify periods of increased crop risk and support locally adapted monitoring and treatment criteria.

## 2. Materials and Methods

### 2.1. Study Site and Rearing Conditions in an Unheated Semi-Outdoor Structure

The study was conducted during the 2024 and 2025 growing seasons at the University of Zagreb Faculty of Agriculture, located next to Maksimir Park in Zagreb, Croatia (45.8286° N, 16.0206° E). Experimental populations of *H*. *halys* were maintained inside an unheated structure without active regulation of temperature or relative humidity. The rearing cages were positioned near entrance doors that remained permanently open throughout the study period, allowing continuous exchange with outdoor air. The insects were therefore exposed to naturally fluctuating ambient temperature, relative humidity, and the natural light–dark cycle, while remaining protected from direct precipitation and solar radiation. These conditions are hereafter referred to as unheated semi-outdoor conditions.

### 2.2. Weather Conditions

Daily mean air temperature and daily mean relative humidity data were obtained from the Zagreb–Maksimir meteorological station of the Croatian Meteorological and Hydrological Service, located near the study site. Monthly mean air temperature and relative humidity were calculated from daily records for January–December 2024 and 2025. For each month, the lowest and highest daily mean air temperatures were also determined. The resulting monthly values were plotted to compare seasonal weather patterns and differences between the two study years.

### 2.3. Degree-Day Calculation

Thermal accumulation was calculated using a lower developmental threshold of 12.2 °C, as previously applied in studies of *H*. *halys* development [15,17,47]. Daily degree-day values were calculated from the daily mean air temperature recorded at the Zagreb–Maksimir meteorological station according to the equation:

DD= Tmean − Tbase

where Tmean is the daily mean air temperature and *T*base is the lower developmental threshold of 12.2 °C.

Daily degree-day values equal to or below zero were set to zero. Cumulative degree-days were calculated from 1 January of each study year and determined for the first recorded occurrence of each phenological event, including the first appearance of overwintered adults, egg masses, individual nymphal instars, and newly emerged adults of successive generations. These January-based cumulative values were used as a calendar-based phenological reference and were not interpreted as thermal requirements for diapause termination or post-diapause development. Degree-day accumulation from the first egg masses to the first adult emergence was additionally calculated for each generation. Degree-day accumulation was used as a descriptive phenological index for comparison with previous European studies.

### 2.4. Establishment and Maintenance of Experimental Populations

Initial adults of *H*. *halys* were collected at the study site in early April of both 2024 and 2025 using pheromone-baited CYMATRAP PRO pyramid traps (GEA S.r.l., Settimo Milanese, Italy). In 2024, 775 overwintered adults (367 females and 408 males) were collected. Of these, 720 individuals were initially allocated to 36 mesh-sided rearing cages (BugDorm, 30 × 30 × 30 cm), with 10 females and 10 males per cage. The remaining adults were maintained separately as reserve individuals and were used only, when necessary, to replace occasional losses during the initial establishment of the colony. No further replacement of adults was performed once the experimental populations were established. In 2025, 200 adults (100 females and 100 males) were allocated to 10 cages using the same standardized sex ratio. The difference in the number of cages between years reflected the number of overwintered adults available for establishing the experimental populations. Consequently, absolute abundance was not used for direct quantitative comparisons between years. The cages were maintained under the unheated semi-outdoor conditions described above. Throughout the rearing period, adults and nymphs were provided with potted bean plants (*Phaseolus vulgaris* L.), fresh carrots, and a commercial trail mix containing almonds, hazelnuts, Brazil nuts, cashews, peanuts, and golden raisins. Water was supplied through moistened sponges. Food and water were replaced twice weekly. When egg masses were detected on bean plants, the entire egg-bearing plant was removed from the parental adult cage and transferred to a designated mesh-sided BugDorm cage for further development. After hatching, the resulting nymphs were maintained separately from the parental adults under the same semi-outdoor conditions and provided with the same food and water sources until adult emergence. Newly emerged adults were sexed and transferred to generation-specific cages, with 10 females and 10 males placed in each cage. They were maintained under the same semi-outdoor conditions with continuous access to food and water and were allowed to feed, mate and undergo reproductive maturation naturally. The onset of the subsequent generation was defined by the first observed oviposition in these cages. Thus, successive generations were physically separated throughout the experiment, and egg masses were assigned to a new generation only when they were produced by adults maintained in the corresponding generation-specific cages. The rearing approach was generally based on that described by Rot et al. [47] but differed in that all egg masses were retained and reared to adulthood, rather than only a selected subset, and newly emerged adults were used to establish the subsequent generation under the same standardized sex ratio.

### 2.5. Monitoring of Seasonal Development and Voltinism

Seasonal development of *H*. *halys* was monitored throughout the 2024 and 2025 growing seasons, from the establishment of the experimental populations in early April until the cessation of developmental and reproductive activity in autumn (October). The cages were inspected daily throughout the rearing period, and adult mortality, mating, oviposition, egg hatching, the appearance of first- to fifth-instar nymphs, and the emergence of new adults were recorded. For each study year, the date of the first observed occurrence of each developmental stage was determined. The onset of oviposition was defined as the date on which the first egg mass was recorded. The first occurrence of each nymphal instar was defined as the date on which individuals of that instar were first observed, whereas the appearance of a new adult generation was defined as the date on which the first adult emerged from the generation-specific developmental cages. The phenological interval from the first recorded egg mass to the first adult emergence within the same generation was calculated in calendar days. Because several egg masses could be produced within a relatively short period, this interval was not interpreted as cohort-specific egg-to-adult developmental time. Individuals originating from eggs laid by post-overwintering females were assigned to the first generation. Subsequent generations were distinguished based on the physical separation of parental adults and their offspring in generation-specific cages, together with the chronological sequence of oviposition, egg hatching, nymphal development, and adult emergence. A generation was considered complete when development from the egg stage through all five nymphal instars to the adult stage was recorded. Voltinism was determined from the number of complete egg-to-adult developmental cycles observed within a single growing season. The first occurrence date of each developmental stage was related to the corresponding cumulative degree-day value calculated as described in Section 2.3.

### 2.6. Overwintering Experiment Under Heated and Unheated Indoor Conditions

Overwintering survival of *H. halys* adults was evaluated under heated and unheated indoor conditions from November 2024 to April 2025. The experiment was initiated on 7 November 2024. Before placement into overwintering shelters, all adults were sexed and assigned to the two treatments. The experimental design generally followed the approaches described by Costi et al. [20] and Rot et al. [47], with modifications related to population size and environmental conditions. In both treatments, adults were placed in wooden overwintering boxes (30 × 20 × 14 cm) filled with layered cardboard to provide suitable hiding sites. The boxes were identical in dimensions and construction in both treatments, and the amount and arrangement of layered cardboard were kept consistent among boxes. Each wooden box was placed inside a mesh cage to prevent adults from dispersing after leaving the overwintering shelter while allowing their emergence to be monitored.

The heated indoor group originated from *H. halys* adults that spontaneously entered a residential building during autumn and selected sheltered sites within the heated interior for overwintering. A total of 1000 of these naturally aggregating adults were transferred to five wooden overwintering boxes containing cardboard refuges and maintained within the same heated environment (approximately 22 °C) until spring. This treatment was intended to represent a heated anthropogenic overwintering refuge rather than natural outdoor diapause conditions. The final survival assessment was conducted on 2 April 2025.

In the unheated treatment, 1200 adults, were distributed among four wooden boxes, with 300 individuals placed in each box. The numbers of adults available for the two overwintering situations differed, resulting in 200 individuals per box in the heated treatment and 300 individuals per box in the unheated treatment; population density was therefore not experimentally standardized between treatments. The boxes were maintained in an unheated indoor facility under naturally fluctuating temperature and relative humidity conditions. The final survival assessment was conducted on 31 March 2025.

At the final assessment, surviving adults were counted. Overwintering survival was calculated separately for each treatment as the percentage of surviving adults relative to the initial number of individuals included in that treatment.

### 2.7. Pheromone-Trap Monitoring

Seasonal population dynamics of *H*. *halys* were monitored in the Maksimir Experimental Orchard of the Department of Pomology, University of Zagreb Faculty of Agriculture. The orchard was located approximately 100 m from the site where the experimental populations were maintained, and seasonal development was monitored. Covering approximately 0.5 ha, the orchard contained several cultivars of apple, pear, sweet cherry, plum, and other fruit species. One pyramid-shaped CYMATRAP PRO trap (GEA S.r.l., Settimo Milanese, Italy) baited with a two-component species-specific aggregation pheromone lure supplied by Trécé Inc. (Adair, OK, USA) was deployed in each study year. The trap was installed in March, before the expected spring emergence of post-overwintering adults, and remained in the orchard throughout the monitoring period. The pheromone lure was replaced on 22.3., 25.5., 29.7. and 12.9. in 2024 and on 20.3., 25.5., 19.7. and 17.9. in 2025. The trap was inspected weekly until late October. At each inspection in 2024, captured adults were counted, while nymphs were classified as second- to fifth-instar nymphs (L2–L5). Nymphal instars were recorded in 2024 as supplementary phenological information but were not a monitoring focus in 2025; therefore, nymphal captures were not compared between years. Because no unbaited control trap was included, nymphal captures were treated as descriptive observations of their presence in and around the trap and were not interpreted as evidence of attraction to the pheromone lure. In 2025, only adult captures were recorded using the same monitoring procedure. Weekly trap catches were used to describe seasonal trap-capture pattern and to compare adult activity between the two study years.

### 2.8. Data Processing and Analysis

Daily meteorological data were used to compare climatic conditions between the two study years. Mean daily air temperature, mean daily relative humidity R, and daily precipitation were analysed separately for each calendar month by comparing the corresponding monthly periods in 2024 and 2025. Temperature and RH are reported as monthly mean ± standard deviation (SD), calculated from daily observations, whereas precipitation is presented as the cumulative monthly total. Differences in daily temperature and RH between corresponding months of the two years were assessed using two-sided Welch’s *t*-tests. This test was selected because it does not assume equal variances between the two groups. The distribution of daily observations was additionally examined for normality using the Shapiro–Wilk test. Because daily precipitation data were strongly non-normal and included a high proportion of days without precipitation, differences in precipitation between corresponding months were assessed using the non-parametric Mann–Whitney U test. To account for multiple testing arising from the 12 month-by-month comparisons, *p*-values were adjusted separately for each meteorological variable using the Holm procedure. Differences were considered statistically significant at an adjusted *p*-value < 0.05. Significance levels were denoted as: * *p*_adj < 0.05, ** *p*_adj < 0.01, and *** *p*_adj < 0.001.

Phenological data were summarized separately for 2024 and 2025. Developmental duration was expressed in calendar days from the first recorded egg mass to the first adult emergence within each generation, and the corresponding cumulative degree-day values were calculated as described in Section 2.3.

Overwintering survival was calculated as the percentage of surviving individuals per cage, with cage considered the experimental unit. Because five cages were available for the heated facility and four for the unheated facility, survival between facilities was compared as an unpaired dataset with unequal sample size. For each facility, mean survival, standard deviation, median and range were calculated. Differences in survival between facilities were analysed using Welch’s two-sample *t*-test, with the Mann–Whitney U test used as a non-parametric comparison. Pooled survival proportions were also calculated from the total number of surviving and initially introduced individuals and compared using a two-proportion test and Fisher’s exact test. Sex composition was expressed as the percentage of males and females among overwintered individuals. Cages without surviving individuals were excluded from sex-ratio calculations. Differences in overall sex composition between facilities were analysed using Fisher’s exact test. Because each thermal regime was represented by a single facility, the cage-level analyses were considered exploratory comparisons of survival patterns between the two overwintering regimes and were not interpreted as a replicated test of the causal effect of temperature.

Weekly pheromone-trap captures were expressed as the number of individuals captured per trap. In 2024, adult captures were summarized as total weekly catches, whereas nymphal captures were grouped by developmental instar (L2–L5). Pheromone-trap catches were standardized as the number of adults captured per trap per day to account for differences in the duration of trapping intervals. For each interval between consecutive trap inspections, mean air temperature, mean relative humidity, and mean daily precipitation were calculated from daily meteorological records. Associations between standardized adult catches and meteorological variables were assessed separately for 2024 and 2025 using Spearman’s rank correlation coefficient.

Statistical analyses were performed in ARM 2026.1. and Python 3.13.5 using SciPy 1.17.0.

## 3. Results

### 3.1. Weather Conditions at Study Area During 2024 and 2025

**Figure 1:**
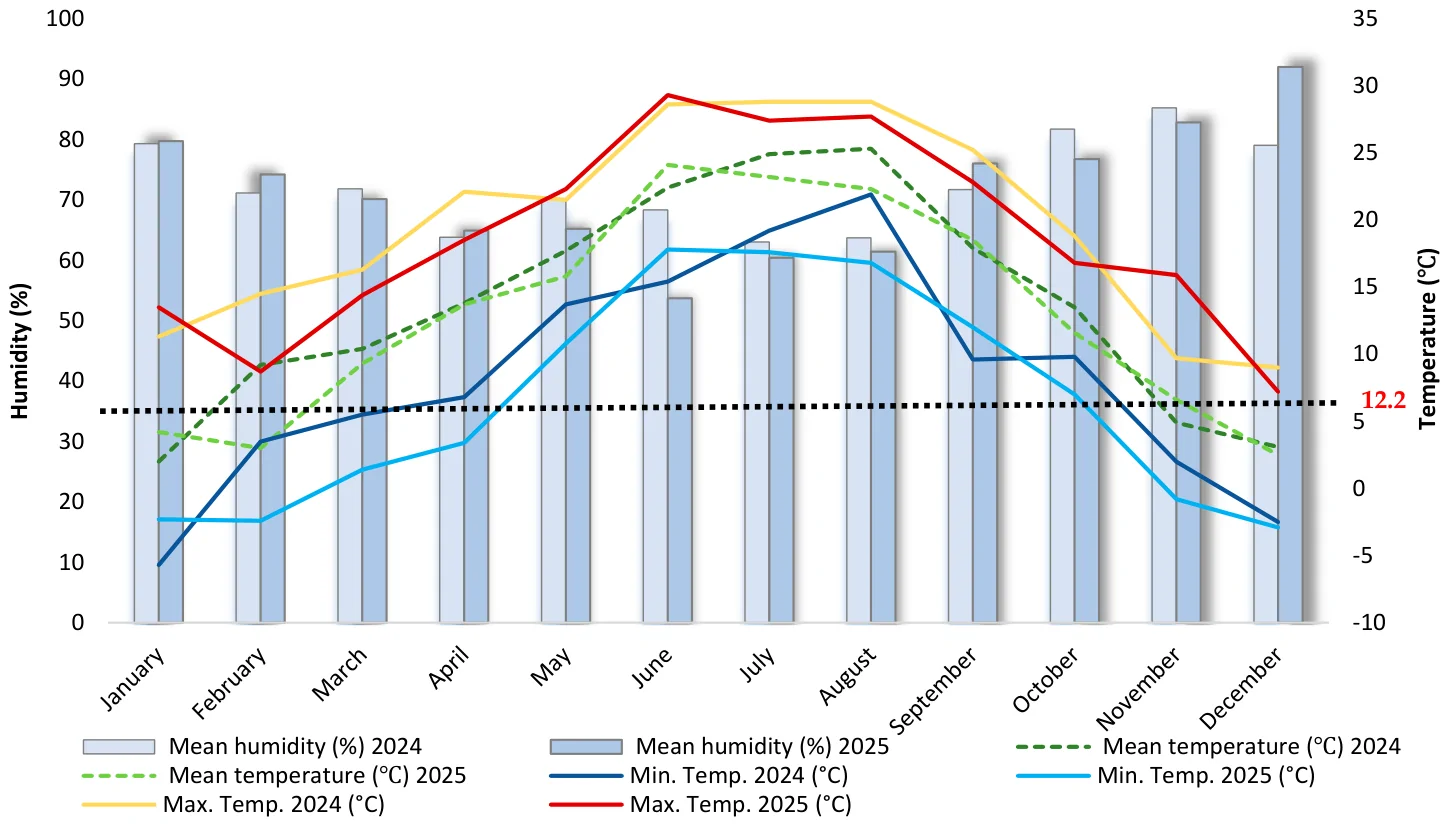
Monthly mean relative humidity and monthly mean, monthly mean minimum, and monthly mean maximum air temperatures recorded at the Zagreb–Maksimir meteorological station in 2024 and 2025 (12.2 °C line is *H. halys* developmental threshold).

Weather conditions showed distinct seasonal patterns between the two study years (Figure 1). In 2024, mean monthly temperatures were higher from February to May and during most of the summer, with the highest monthly mean temperature recorded in August (25.3 °C). In 2025, spring temperatures were generally lower, followed by a rapid increase in June, when the highest monthly mean temperature of that year was recorded (24.1 °C). Summer temperatures subsequently declined in July and August 2025. Monthly mean minimum temperatures were generally higher during summer 2024, particularly in August (21.9 °C compared with 16.8 °C in 2025), indicating warmer night-time conditions. Relative humidity also differed seasonally between years, with particularly dry conditions observed in June 2025.

Statistical comparisons confirmed significant interannual differences for several months (Table 1). Mean daily temperature was significantly higher in February 2024 than in February 2025 (9.16 ± 2.82 vs. 2.96 ± 2.70 °C; *p* < 0.001). Significantly higher temperatures were also recorded in August 2024 (25.26 ± 2.04 vs. 22.30 ± 2.97 °C; *p* < 0.001) and October 2024 (13.50 ± 2.25 vs. 11.57 ± 2.56 °C; *p* = 0.027). Relative humidity was significantly lower in June 2025 than in June 2024 (53.7 ± 6.6 vs. 68.3 ± 8.0%; *p* < 0.001), accompanied by substantially lower monthly precipitation (9.9 vs. 79.9 mm; *p* = 0.007). In contrast, relative humidity was significantly higher in December 2025 than in December 2024 (92.3 ± 5.1 vs. 79.3 ± 10.9%; *p* < 0.001). No other month-to-month comparisons remained significant after adjustment for multiple comparisons.

Monthly mean minimum and maximum temperatures also differed in their seasonal patterns between the two years (Figure 1). During winter, January 2024 had a lower mean minimum temperature than January 2025 (−5.7 vs. −2.3 °C), whereas February 2025 was colder than February 2024, with both lower minimum (−2.4 vs. 3.5 °C) and maximum temperatures (8.7 vs. 14.5 °C). From March to May, minimum temperatures were consistently higher in 2024, while maximum temperatures were also generally higher, except in May, when the maximum temperature was slightly higher in 2025 (22.3 vs. 21.5 °C). In June, both minimum and maximum temperatures were higher in 2025 (17.8 and 29.3 °C, respectively) than in 2024 (15.4 and 28.6 °C). This pattern reversed during July and August, when both minimum and maximum temperatures were higher in 2024, with the largest summer difference in minimum temperature recorded in August (21.9 vs. 16.8 °C). In September and October, maximum temperatures remained higher in 2024, whereas minimum temperatures were higher in September 2025 but lower again in October 2025. November showed the largest annual difference in maximum temperature, reaching 15.9 °C in 2025 compared with 9.7 °C in 2024, despite a lower minimum temperature in 2025 (−0.8 vs. 2.0 °C). In December, both minimum and maximum temperatures were slightly lower in 2025. Overall, the two years differed not only in mean temperature but also in the seasonal distribution of minimum and maximum temperatures, indicating distinct patterns of winter cold, spring warming, summer heat, and autumn cooling.

### 3.2. Overwintering Survival Under Heated and Unheated Indoor Conditions

Overwintering survival was numerically higher in the heated facility than in the unheated facility when all available cages were included. Mean survival across cages was 9.40% in the heated facility and 7.83% in the unheated facility, corresponding to a difference of 1.57 percentage points. Survival ranged from 5.50% to 15.50% in the heated facility and from 0.00% to 11.67% in the unheated facility. Because one heated cage had no corresponding unheated counterpart, the comparison was analysed as an unpaired comparison with unequal sample size. Neither Welch’s two-sample *t*-test (*t* = 0.49, df = 5.15, *p* = 0.647) nor the Mann–Whitney U test (U = 10.0, *p* = 1.000) indicated a statistically significant difference between facilities.

When survival was additionally examined as pooled proportions, 94 of 1000 individuals survived in the heated facility, compared with 94 of 1200 individuals in the unheated facility. This corresponded to survival rates of 9.40% and 7.83%, respectively, and a risk ratio of 1.20. However, the difference was not statistically significant in either the two-proportion test (*p* = 0.191) or Fisher’s exact test (*p* = 0.194). These pooled results should be interpreted cautiously because individuals within the same cage are not fully independent experimental units.

The sex composition of overwintered individuals showed a female-biased pattern in both facilities. Across all available cages, females represented 58.5% of overwintered individuals in the heated facility and 68.1% in the unheated facility. Although the female bias was more pronounced in the unheated facility, the difference in sex composition between facilities was not statistically significant (Fisher’s exact test: *p* = 0.226).

**Figure 2:**
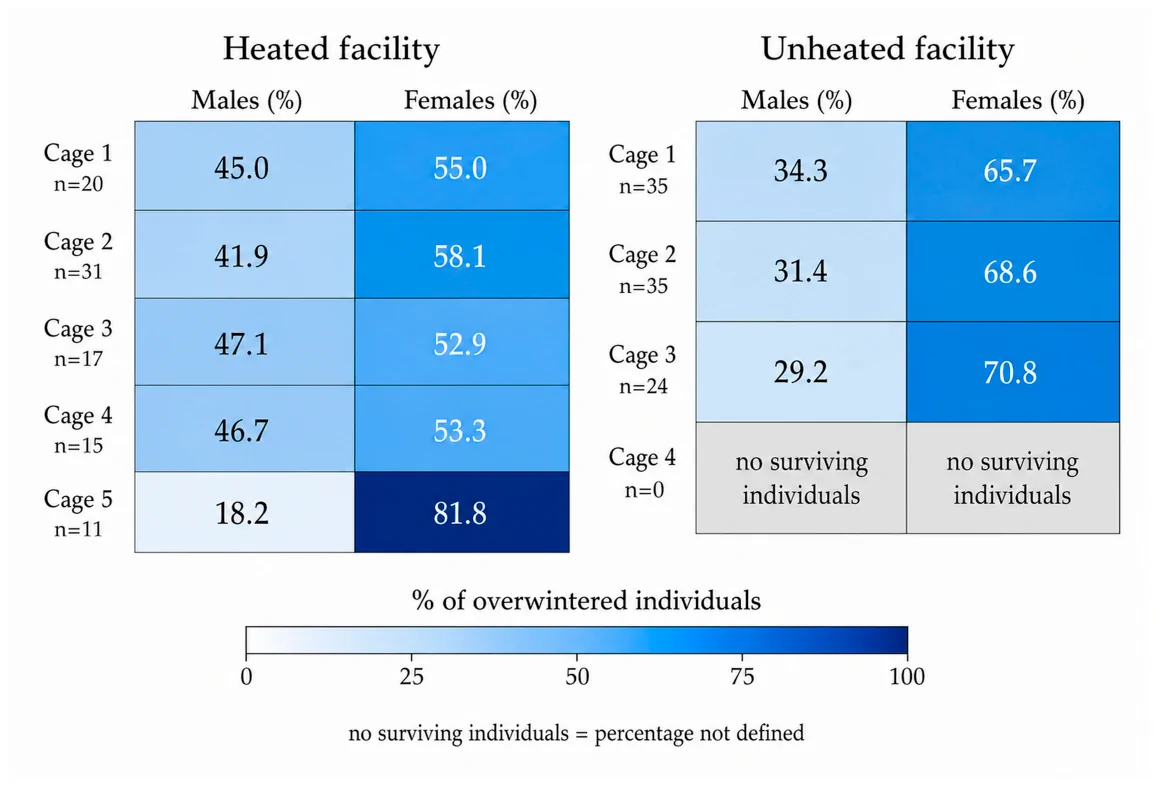
Sex composition of overwintered individuals by cage and facility. Cell values represent the percentage of males and females among overwintered individuals within each cage. Sample size (n) below each cage label indicates the total number of individuals that survived overwintering in that cage.

The heat map at Figure 2 presents the sex composition of overwintered individuals separately for each cage and facility. Percentages were calculated within each cage as the proportion of males or females among the total number of individuals that survived overwintering. In the heated facility, females represented 52.9–81.8% of overwintered individuals, with the strongest female bias recorded in Cage 5. In the unheated facility, females represented 65.7–70.8% of overwintered individuals in cages where survival was recorded. In Cage 4 of the unheated facility, no individuals survived.

### 3.3. Seasonal Phenology and Voltinism

**Figure 3:**
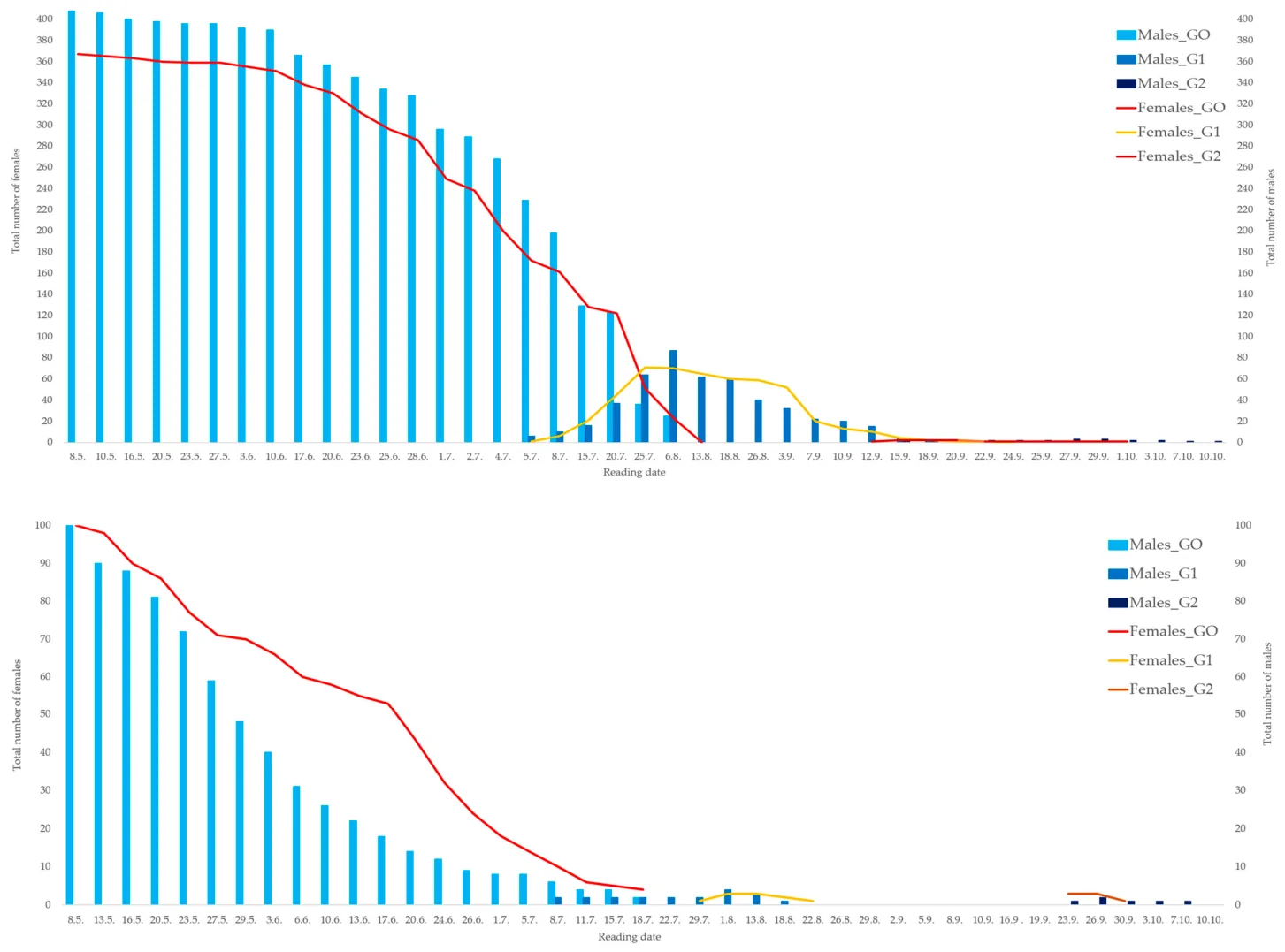
Seasonal development of adult *H. halys* under unheated semi-outdoor conditions in 2024 (upper graph) and in 2025 (lower graph). Temporal overlap among developmental stages shown in the figure reflects their concurrent seasonal occurrence in separate generation-specific rearing cages and does not indicate mixing of generations within cages.

Two complete egg-to-adult generations of *H*. *halys* were recorded under unheated semi-outdoor conditions in both 2024 and 2025. In each year, adults collected after overwintering produced a first generation during spring and early summer, followed by a second generation that completed development during late summer and early autumn (Figure 3). Developmental stages showed temporal overlap within the seasonal phenological record, particularly from June onwards in G1 and during August and September in G2. Because generations were maintained in separate generation-specific cages, this temporal overlap did not affect generational assignment.

#### 3.3.1. Seasonal Development in 2024

The first overwintered adults were recorded on 5 April 2024, after which individuals were collected to establish the experimental population and the seasonal development experiment was initiated. Their abundance gradually declined during spring and early summer, with a more pronounced decrease from June onwards (Figure 3). The first egg masses of G1 were recorded on 10 May. First-instar nymphs appeared on 27 May, followed by L2 on 3 June, L3 on 10 June, L4 on 17 June, and L5 on 1 July (Table 2).

The first G1 adults emerged on 5 July, completing the first egg-to-adult developmental cycle in 56 days (Table 2). First-instar nymphs were the most abundant stage of G1 and reached their maximum abundance in early June. The abundance of subsequent nymphal instars increased sequentially, with peaks occurring later in the season. Eggs, several nymphal instars, and newly emerged adults overlapped from June into July. G1 adults remained present during July and August, while their offspring initiated the second generation (Figure 3).

**Figure 4:**
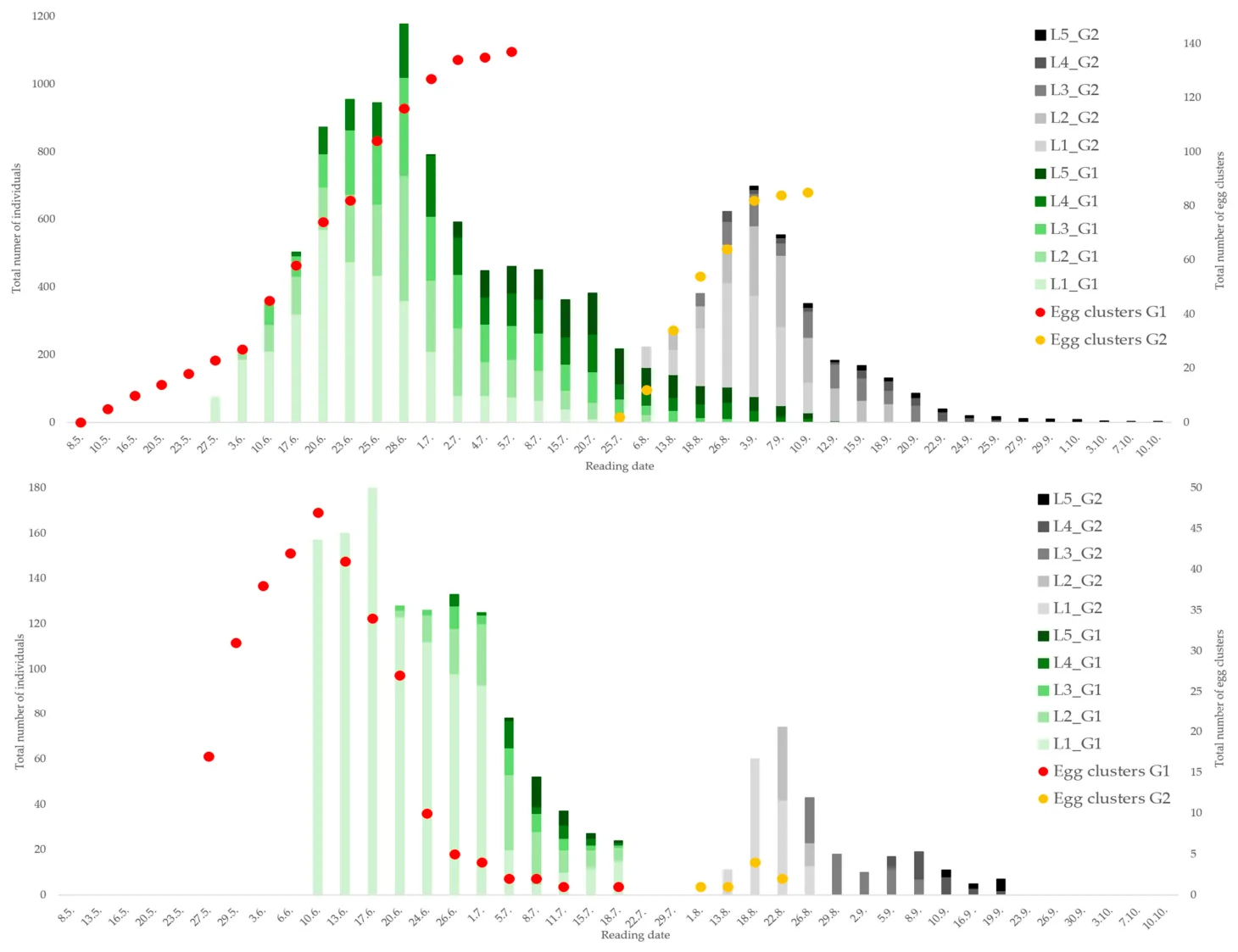
Seasonal development of *H. halys* nymphs and eggs under unheated semi-outdoor conditions in 2024 (upper graph) and in 2025 (lower graph). Temporal overlap among developmental stages shown in the figure reflects their concurrent seasonal occurrence in separate generation-specific rearing cages and does not indicate mixing of generations within cages.

The first G2 egg masses were recorded on 25 July. First-instar nymphs appeared on 6 August, followed by L2 on 13 August, L3 on 18 August, L4 on 26 August, and L5 on 3 September. The first G2 adults emerged on 12 September, corresponding to an egg-to-adult developmental period of 49 days (Figure 4, Table 2). The highest abundance within G2 was recorded for L1 during the second half of August, followed by a sequential increase and decline in the abundance of later instars. Eggs, nymphs, and adults of G2 overlapped during late August and September (Figure 3 and Figure 4). Reproductive activity declined during September, while seasonal activity ended in October (Table 2).

#### 3.3.2. Seasonal Development in 2025

The first overwintered adults were recorded on 2 April 2025 after which individuals were collected to establish the experimental population and the seasonal development experiment was initiated. Their abundance declined progressively during spring and early summer, with only a small number of adults remaining by July (Figure 3, Table 2). The first G1 egg masses were recorded on 27 May, 17 days later than in 2024. First-instar nymphs appeared on 10 June, followed by L2 on 17 June, L3 on 20 June, L4 on 26 June, and L5 on 5 July. The first G1 adults emerged on 8 July, completing development from the first egg masses to the first adults in 42 days (Table 2).

The abundance of G1 egg masses increased during May and reached its maximum near the end of the month. First-instar nymphs were the most abundant immature stage and reached their highest abundance in late May and early June. Later nymphal instars occurred at lower abundances and overlapped during June and July. Newly emerged G1 adults were recorded from early July onwards (Figure 3).

The first G2 egg masses were observed on 1 August. First-instar nymphs appeared on 13 August, followed by L2 on 22 August, L3 on 26 August, L4 on 5 September, and L5 on 10 September. The first G2 adults emerged on 23 September, corresponding to an egg-to-adult developmental period of 53 days (Table 2). The abundance of G2 was lower than that of G1. First-instar nymphs reached their highest abundance in August, followed by the sequential occurrence of later instars during late August and September. Reproductive activity declined during September, and seasonal activity ended in October (Figure 4; Table 2).

#### 3.3.3. Interannual Comparison of Developmental Duration and Degree-Day Accumulation (DD_12.2_)

The first overwintered adults were recorded on 5 April 2024 and 2 April 2025, corresponding to 31.6 and 11.0 cumulative DD_12.2_, respectively. The first G1 egg masses were recorded on 10 May 2024 at 158.4 DD_12.2_ and on 27 May 2025 at 171.3 DD_12.2_ (Table 2).

Although G1 oviposition began 17 days later in 2025, the first G1 adults emerged only three days later than in 2024. Development from the first egg masses to the first adults lasted 56 days in 2024 and 42 days in 2025. The corresponding thermal accumulations were 468.5 DD_12.2_ in 2024 and 480.1 DD_12.2_ in 2025 (Table 2).

The first G2 egg masses were recorded on 25 July 2024 and 1 August 2025, at 908.7 and 906.9 cumulative DD_12.2_, respectively. The first G2 adults emerged on 12 September 2024 at 1471.4 DD_12.2_ and on 23 September 2025 at 1381.0 DD_12.2_. Development from the first G2 egg masses to the first adults lasted 49 days in 2024 and 53 days in 2025, corresponding to thermal accumulations of 562.7 and 474.1 DD_12.2_, respectively (Table 2).

Across both years and generations, egg-to-adult development required between 468.5 and 562.7 DD_12.2_. Two complete generations were recorded in each study year, confirming bivoltine development under the study conditions.

### 3.4. Seasonal Trap-Capture Pattern Based on Pheromone-Trap Captures

**Figure 5:**
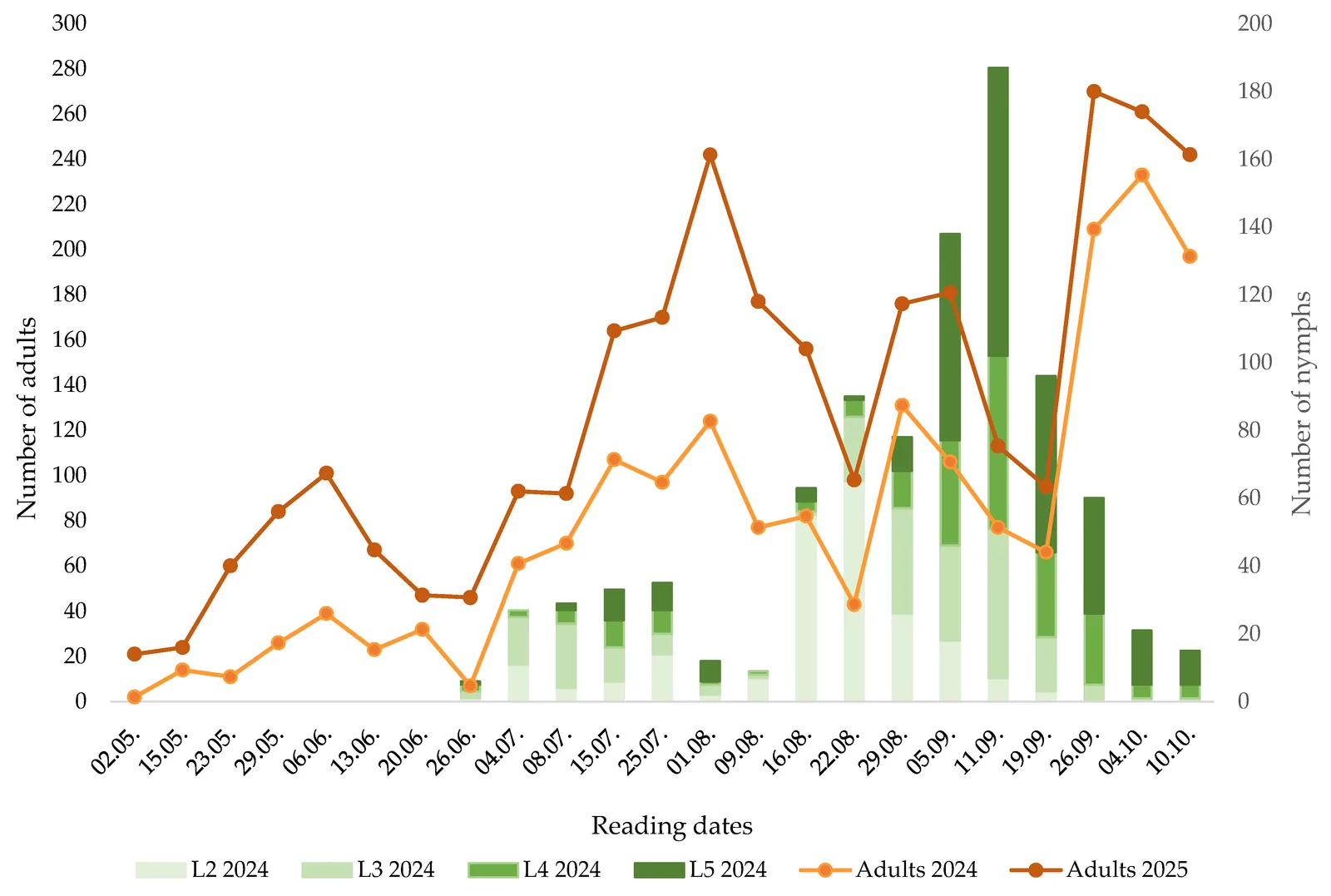
Weekly captures of *H. halys* adults during 2024 and 2025 and weekly captures of second- to fifth-instar nymphs (L2–L5) in pheromone-baited traps during 2024 in the Maksimir Experimental Orchard, Zagreb, Croatia.

Adults were captured in both study years, although the timing and magnitude of captures differed (Figure 5). In 2024, adult captures were low during May and June and increased from July onwards. A first marked increase was recorded in late July and early August, followed by a temporary decline during the second half of August and early September. Captures then increased sharply in late September and reached their annual maximum of 232 adults on 4 October. High numbers were also recorded on 26 September and 10 October.

In 2025, adult captures increased earlier in the season than in 2024. Numbers rose during May and June and reached their highest level in early August, at approximately 120 adults per trap. Captures subsequently declined, although smaller increases were recorded in late August and early September. From mid-September onwards, adult captures remained considerably lower than those recorded during the same period in 2024. The pronounced autumn peak observed in 2024 was not recorded in 2025 (Figure 5).

Nymphs (L2–L5) were recorded in the pheromone trap only in 2024. L2 nymphs reached their highest abundance in late August, with approximately 65 individuals captured on 22 August. Captures of L3 and L4 increased from late August and reached their highest values in September. L5 nymphs showed the most pronounced late-season increase, rising sharply in early September and reaching a maximum of approximately 85 individuals on 11 September. L5 captures remained comparatively high until late September and were still recorded in October. Several nymphal instars were captured simultaneously from late August to September (Figure 5). These nymphal captures indicate seasonal presence near the trap but do not allow conclusions regarding attraction to the pheromone lure.

Spearman’s rank correlation analysis showed no significant associations between standardized adult pheromone-trap catches and meteorological conditions recorded during the corresponding trapping intervals (Table 3). In 2024, correlations with mean temperature (ρ = 0.115, *p* = 0.603), relative humidity (ρ = −0.103, *p* = 0.639), and mean daily precipitation (ρ = −0.090, *p* = 0.684) were weak and non-significant. Similarly, no significant relationships were detected in 2025 for mean temperature (ρ = 0.208, *p* = 0.342), relative humidity (ρ = −0.002, *p* = 0.993), or precipitation (ρ = 0.281, *p* = 0.194). These results indicate that short-term variation in adult trap catches could not be explained by any single meteorological variable considered independently.

## 4. Discussion

### 4.1. Seasonal Phenology and Potential for Two Generations

The present study followed physically separated successive generations of *Halyomorpha halys* from oviposition through all five nymphal instars to adult emergence and documented the completion of two egg-to-adult developmental cycles in both 2024 and 2025. Under the unheated semi-outdoor conditions used in this study, local temperature and photoperiod therefore provided sufficient seasonal conditions for the completion of a second generation. Because food was continuously available and several ecological constraints occurring in unrestricted field populations were reduced, these results are best interpreted as evidence of the developmental potential for two generations at the study site, rather than as confirmation that two generations are consistently completed by free-living populations throughout continental Croatia. The observed pattern is consistent with studies from warmer parts of southern and central Europe. Costi et al. [20] documented two generations per year under temperate–Mediterranean conditions in northern Italy, while Rot et al. [47] recorded two overlapping generations in western Slovenia, with first-generation adults emerging during summer and second-generation adults appearing from mid-September onwards. Seasonal patterns compatible with two generations have also recently been reported in northern Greece [49]. In contrast, pheromone-based monitoring in Ljubljana indicated predominantly univoltine development [48], illustrating the regional variability of *H. halys* phenology. Such differences may reflect local temperature regimes, photoperiod, host availability and landscape conditions [11,18,19,21,25], but also the methodology used to identify generations. In the present study, G0, G1 and G2 were physically separated, so the generational identity of eggs, nymphs and newly emerged adults remained known even when their seasonal occurrence overlapped. This provides direct evidence that two complete developmental cycles were possible under the conditions experienced at the Zagreb study site.

The timing of development was similar for several key events despite differences between years. G1 adults first emerged on 5 July 2024 and 8 July 2025, whereas G2 adults appeared on 12 September 2024 and 23 September 2025. The later appearance of G2 adults in 2025 followed the later initiation of G2 oviposition and illustrates greater interannual variability toward the end of the growing season. Such flexibility is consistent with the strong influence of temperature, photoperiod and seasonal physiological responses on *H. halys* development and diapause induction reported previously [11,12,13,14,18,19].

### 4.2. Thermal Accumulation and Interannual Weather Variability

Thermal accumulation provided a useful quantitative framework for comparing developmental events between years and with previous European observations. From the first recorded egg masses to the first adult emergence, G1 accumulated 468.5 DD_12.2_ in 2024 and 480.1 DD_12.2_ in 2025, whereas G2 accumulated 562.7 and 474.1 DD_12.2_, respectively. These values broadly correspond to the approximately 531 and 545 DD_12.2_ reported for the first and second generations in western Slovenia [47]. The similarity of G1 thermal accumulation between years is particularly notable because oviposition began 17 days later in 2025, while the first G1 adults appeared only three days later than in 2024. Thus, the shorter observed phenological interval in 2025 coincided with the warmer conditions recorded during early summer.

The larger interannual variation observed during G2 also indicates that thermal accumulation should be interpreted together with seasonal timing. Development of the second generation occurs as photoperiod shortens and reproductive diapause approaches, when temperature is only one component of the seasonal response [11,12,13,14,18,19]. This is consistent with recent validation of *H. halys* phenology models across different climatic regions, which showed that predictions based on thermal accumulation may vary in performance among environments [50]. Degree-days therefore remain valuable for regional phenological forecasting, particularly when interpreted together with locally observed developmental stages.

In the present study, DD_12.2_ values were used as comparative phenological indicators rather than as newly estimated physiological developmental constants. January-based cumulative DD values provide a common calendar reference for seasonal events, while generation-specific values from first oviposition to first adult emergence more directly describe thermal accumulation during immature development. This distinction is important because diapause termination and reproductive activation are also controlled by photoperiod and physiological state [13,14,18,30]. Taken together, the results support the use of locally calibrated thermal accumulation as one component of phenological monitoring rather than as a stand-alone predictor of population development.

### 4.3. Stage Overlap, Pheromone-Trap Activity and Implications for Monitoring

Temporal overlap among developmental stages was a prominent feature of the seasonal pattern. During summer and early autumn, eggs, several nymphal instars and adults were present concurrently across the generation-specific cages. Similar overlap has been described in northern Italy and western Slovenia [20,47]. From an IPM perspective, this means that calendar date alone provides limited information on the stage composition of the population. Direct observations of eggs and young nymphs are particularly informative because they identify the establishment of a new generation before later, more mobile stages become dominant.

Pheromone-trap monitoring of the naturally occurring population in the nearby orchard was independent of the experimentally reared cohorts and provided a complementary measure of site-specific adult activity. Adult captures differed between years: in 2024, the strongest increase occurred in late September and the annual maximum of 232 adults was recorded on 4 October, whereas in 2025 the principal peak occurred earlier, in early August. Such differences illustrate that pheromone-trap catches integrate abundance, movement and behavioural responsiveness to the lure rather than representing population density alone [37,38,39,41,42,51]. This interpretation is further supported by the present correlation analysis. Standardized adult catches were not significantly associated with mean temperature, relative humidity or precipitation during the corresponding trapping intervals in either year. Short-term trap-catch variation therefore could not be attributed to any single meteorological variable examined independently.

The observed phenology provides several practical monitoring windows. Monitoring of adults should begin with spring activity in early April. From May through June, plant inspections should increasingly focus on egg masses and young nymphs, which indicate establishment of G1. The emergence of G1 adults in early July, followed by G2 oviposition from late July to early August, identifies a second important period for intensified stage-specific monitoring. Monitoring should then continue through September and into harvest in late-ripening crops, because late nymphal instars and G2 adults remain present during this period. Rather than applying a universal pheromone-trap threshold, local decisions should combine adult captures with direct plant inspection, crop phenology and evidence of crop injury [40,41,42,46]. This approach is particularly relevant in heterogeneous orchard landscapes, where movement between cultivated and non-crop hosts can strongly influence local *H. halys* activity [9,22,25,26].

### 4.4. Overwintering Survival in Anthropogenic Refuges

Overwintering survival was low under both anthropogenic refuge conditions examined, with mean cage-level survival of 9.40% in the heated facility and 7.83% in the unheated facility. The difference between facilities was not statistically supported (Welch’s test, *p* = 0.647), and pooled survival proportions likewise did not differ significantly. The two regimes should therefore be interpreted as contrasting anthropogenic overwintering scenarios, rather than as a replicated experiment isolating the causal effect of temperature. Stocking density also differed between facilities, further precluding attribution of survival differences specifically to thermal regime. This distinction is ecologically relevant because *H. halys* commonly enters buildings and other human-made structures during its autumn migration and may remain within such shelters throughout winter [31,32].

Previous studies show that overwintering success in *H. halys* varies substantially among populations, years and environmental conditions [20,27,28,33,34,35,47,52,53]. Recent multi-year comparisons under laboratory and sheltered semi-field conditions likewise showed substantial variation in overwintering mortality among cohorts and years, with no simple or consistent relationship between environmental regime and survival [53]. Accordingly, survival observed in the present sheltered anthropogenic refuges should not be extrapolated directly to natural outdoor overwintering conditions. Temperature, humidity, physiological condition, diapause status and refuge characteristics have all been identified as relevant components of overwintering biology [27,28,29,30,31,32,33,34,35,36]. These mechanisms were not measured separately in the present study, so the observed mortality is interpreted at the level of the refuge conditions rather than attributed to a specific physiological process. The survival recorded here was lower than that reported in western Slovenia [47], consistent with the substantial variability in overwintering outcomes reported among populations and experimental settings. Females represented the majority of survivors in both facilities, which is biologically relevant because they constitute the reproductive component capable of contributing to population renewal in spring. Spring emergence and subsequent reproduction nevertheless represent additional stages in the seasonal cycle, and previous work has shown that emergence from overwintering shelters may extend over a prolonged period [30,35,36,54].

### 4.5. Study Scope and Future Perspectives

The present study provides a detailed two-year phenological dataset for *H. halys* at a single continental Croatian site under standardized semi-outdoor conditions. This design enabled successive generations to be followed consistently and allowed developmental events to be related to local thermal accumulation. At the same time, the standardized rearing conditions should be considered when extrapolating the results to unrestricted field populations. In particular, continuous availability of a mixed diet may have reduced variation associated with natural host quality, which is known to influence nymphal survival and developmental duration in *H. halys* [55]. Differences in initial population size between years also limit direct comparison of absolute abundance, while first-occurrence dates should be interpreted as indicators of phenological onset rather than population-level mean developmental times. Degree-day values were derived from daily mean temperatures recorded at a nearby meteorological station and are therefore most appropriately interpreted as local phenological estimates rather than precise physiological constants. Similarly, the overwintering experiment represents survival under two anthropogenic refuge conditions, while pheromone monitoring provides a site-specific record of seasonal adult activity. Despite these considerations, the study establishes a coherent local framework linking cohort development, thermal accumulation, overwintering survival, and seasonal trap activity. Future multi-year and multi-site studies incorporating direct microclimate measurements, replicated trapping, natural host plants, and more detailed demographic measurements would further strengthen and extend this framework.

## 5. Conclusions

The present study demonstrated that two complete *H. halys* generations could develop under the local conditions at the Zagreb study site. G1 adults emerged in early July in both years, G2 oviposition began from late July to early August, and G2 adults appeared from mid- to late September. Generation-specific development from the onset of oviposition to first adult emergence corresponded to 468.5–562.7 DD_12.2_, providing locally relevant phenological benchmarks for monitoring. These results identify practical periods for IPM surveillance: adult monitoring should begin in early April, plant inspections for egg masses and young nymphs should be intensified during May and June, and a second period of intensified monitoring should begin in late July–early August as G2 develops and continue through September in crops remaining susceptible late in the season. Pheromone-trap catches should be used primarily as indicators of adult activity and interpreted together with direct stage-specific observations. Overwintering survival of 7.8–9.4% under the two anthropogenic refuge conditions further indicates that seasonal population development reflects both the capacity to complete successive generations and the number of adults successfully entering the following reproductive season. Overall, the study provides a locally grounded phenological framework for more precisely timed monitoring and IPM decisions for *H*. *halys* under temperate continental conditions.

## Figures and Tables

**Table 1:** Monthly meteorological conditions at Zagreb–Maksimir in 2024 and 2025 and statistical comparison between years.

Month	Mean Temperature 2024 (°C)	Mean Temperature 2025 (°C)	Sig.	Mean RH 2024 (%)	Mean RH 2025 (%)	Sig.	Precipitation 2024 (mm)	Precipitation 2025 (mm)	Sig.
January	1.97 ± 4.47	4.22 ± 5.55	ns	79.3 ± 8.7	79.7 ± 11.5	ns	64.6	56.9	ns
February	9.16 ± 2.82	2.96 ± 2.70	***	71.1 ± 9.5	74.2 ± 10.4	ns	30.1	57.6	ns
March	10.45 ± 2.91	9.27 ± 3.56	ns	71.8 ± 10.9	70.1 ± 12.2	ns	95.0	92.9	ns
April	13.85 ± 5.17	13.68 ± 3.77	ns	63.8 ± 12.0	64.9 ± 10.9	ns	43.3	46.8	ns
May	17.69 ± 1.78	15.81 ± 3.18	ns	70.1 ± 10.7	65.2 ± 11.0	ns	86.8	57.3	ns
June	22.38 ± 3.50	24.08 ± 2.75	ns	68.3 ± 8.0	53.7 ± 6.6	***	79.9	9.9	**
July	24.93 ± 2.86	23.24 ± 3.16	ns	63.0 ± 7.6	60.4 ± 12.6	ns	120.1	104.0	ns
August	25.26 ± 2.04	22.30 ± 2.97	***	63.7 ± 5.9	61.4 ± 11.0	ns	108.5	72.7	ns
September	17.94 ± 4.68	18.49 ± 2.75	ns	71.7 ± 9.6	76.0 ± 8.1	ns	167.4	51.0	ns
October	13.50 ± 2.25	11.57 ± 2.56	*	81.7 ± 8.5	76.7 ± 9.3	ns	96.9	104.8	ns
November	5.44 ± 2.78	6.61 ± 4.56	ns	83.8 ± 9.1	82.8 ± 8.8	ns	79.6	99.5	ns
December	3.11 ± 2.50	2.50 ± 2.27	ns	79.3 ± 10.9	92.3 ± 5.1	***	40.9	46.0	ns

Temperature and relative humidity (RH) are presented as mean ± standard deviation (SD) of daily observations, whereas precipitation is presented as the monthly cumulative total. Statistical significance refers to comparisons between the corresponding months of 2024 and 2025. Temperature and RH were compared using two-sided Welch’s *t*-tests, while daily precipitation was compared using the Mann–Whitney U test. *p*-values were adjusted separately for the 12 monthly comparisons within each meteorological variable using the Holm procedure. ns, not significant; *, *p*_adj < 0.05; **, *p*_adj < 0.01; ***, *p*_adj < 0.001.

**Table 2:** First occurrence of phenological events in *H. halys* during 2024 and 2025, corresponding cumulative degree-day accumulation above 12.2 °C, and published degree-day ranges.

Phenological Event	Observation (2024)	Observation (2025)	2024 Σ Degree-Days (DD_12.2_, Observed)	2025 Σ Degree-Days (DD_12.2_, Observed)	Published Degree-Day Thresholds (DD_12.2_) *
First overwintered adults +	5 April	2 April	31.6 ↑	11.0 ↔	1–11
First egg masses (Gen 1)	10 May	27 May	158.4 ↔	171.3 ↔	150–180
First instar (L1, Gen 1)	27 May	10 June	259.1 ↔	311.8 ↑	240–270
Second instar (L2, Gen 1)	3 June	17 June	298.1 ↔	383.1 ↑	280–320
Third instar (L3, Gen 1)	10 June	20 June	371.3 ↔	416.6 ↑	350–390
Fourth instar (L4, Gen 1)	17 June	26 June	419.1 ↔	499.7 ↑	400–440
Fifth instar (L5, Gen 1)	1 July	5 July	594.5 ↔	621.8 ↑	580–620
First adults (Gen 1)	5 July	8 July	626.9 ↔	651.4 ↔	620–660
First egg masses (Gen 2)	25 July	1 August	908.7 ↔	906.9 ↔	880–950
First instar (L1, Gen 2)	6 August	13 August	1036.5 ↔	1035.4 ↔	1000–1070
Second instar (L2, Gen 2)	13 August	22 August	1131.9 ↔	1135.7 ↔	1100–1180
Third instar (L3, Gen 2)	18 August	26 August	1209.6 ↔	1160.9 ↓	1180–1260
Fourth instar (L4, Gen 2)	26 August	5 September	1308.9 ↔	1254.9 ↓	1280–1360
Fifth instar (L5, Gen 2)	3 September	10 September	1397.4 ↔	1294.5 ↓	1380–1460
First adults (Gen 2) **	12 September	23 September	1471.4 ↔	1381.0 ↓	1450–1550

* Published DD_12.2_ thresholds are literature values (Rot et al. [47], Bohinc [48]) shown for comparison. ** Late instars and emerging adults overlapped in Gen 2, reflecting partial generational overlap under field conditions; + first overwinters adults observed on pheromone traps. ↔ = observed DD value within the published threshold range; ↑ = observed DD value above the published range; ↓ = observed DD value below the published range.

**Table 3:** Spearman rank correlations between standardized adult *Halyomorpha halys* pheromone-trap catches and meteorological variables during corresponding trapping intervals in 2024 and 2025.

Year	Meteorological Variable	Spearman’s ρ	*p*-Value
2024	Mean temperature	0.115	0.603
2024	Mean relative humidity	−0.103	0.639
2024	Mean daily precipitation	−0.090	0.684
2025	Mean temperature	0.208	0.342
2025	Mean relative humidity	−0.002	0.993
2025	Mean daily precipitation	0.281	0.194

Trap catches were standardized as the number of adults captured per trap per day. Meteorological variables were calculated for the corresponding interval between consecutive trap inspections.

## Data Availability

The original contributions presented in this study are included in the article. Further inquiries can be directed to the corresponding authors.
